# Acute-on-Chronic Liver Failure: Steps Towards Consensus

**DOI:** 10.3390/diagnostics15060751

**Published:** 2025-03-17

**Authors:** Loredana Gabriela Goran, Florina Alexandra Liţă (Cofaru), Carmen Fierbinţeanu-Braticevici

**Affiliations:** 1 Emergency University Hospital Bucharest, Carol Davila University of Medicine and Pharmacy, 020021 Bucharest, Romania; florina.cofaru@drd.umfcd.ro (F.A.L.); carmen.fierbinteanu@umfcd.ro (C.F.-B.); 2 Internal Medicine II and Gastroenterology Department, University Emergency Hospital Bucharest, 050098 Bucharest, Romania; 3 Emergency Department, University Emergency Hospital Bucharest, 050098 Bucharest, Romania

**Keywords:** acute-on-chronic liver failure, liver failure, organ failure management, liver transplant

## Abstract

Acute-on-chronic liver failure (ACLF) is a clinical syndrome characterized by organ failure and high short-term mortality. Since its first definition in 2013, many international organizations have defined this syndrome and, till now, there has been no agreement regarding definitions and diagnostic criteria. Although the precise mechanism of ACLF is unknown, precipitant factors and the systemic inflammation response play a major role. Specific management of this high-mortality syndrome is still under development, but a general consensus in the diagnosis and management of ACLF is needed.

## 1. Introduction

Acute-on-chronic liver failure (ACLF) is a syndrome characterized by a severe form of acutely decompensated cirrhosis with manifestations of organ system failure (s) and high short-term mortality [1]. Since the first definition of ACLF in 2009, made by the Asian Pacific Association for the Study of the Liver (APASL) [2], up to 13 definitions of ACLF have been reported [3].

APASL defines ACLF as an acute hepatic insult characterized by jaundice (bilirubin ≥ 5 mg/dL) and coagulopathy (international normalized ratio [INR] ≥ 1.5 or prothrombin activity < 40%) complicated by ascites and/or hepatic encephalopathy in less than 4 weeks in a patient with prior diagnosed or undiagnosed chronic liver disease or cirrhosis and a high mortality rate at 28 days [4]. The APASL definition of ACLF does not require the presence of extrahepatic organ failure for the diagnosis.

The North American Consortium for the Study of End-Stage Liver Disease (NACSELD) includes the presence of at least two severe extrahepatic organ failures like shock, hepatic encephalopathy grade III or IV, mechanical ventilation, or renal replacement therapy in order to define ACLF [5].

The European Association for the Study of the Liver-Chronic Liver Failure Consortium (EASL-CLIF-C) defined ACLF as an acute decompensation in patients with cirrhosis, with the presence of organ failure (liver, kidney, brain, respiratory system, circulation, and coagulation) and an associated 28-day mortality rate of 20% or more [1].

Despite the variability of definitions, the common factor of ACLF patients remains the poor prognosis. Also, the lack of consistency regarding the definition of ACLF brings up problems related to the diagnosis and management of these patients. The main diagnostic criteria of ACLF from NACSELD, EASL-CLIF, and APASL are summarized in Table 1.

A meta-analysis that used the EASL-CLIF criteria for the definition of ACLF, reveals the global burden of this syndrome, with a worldwide prevalence of 35% amid cirrhotic patients admitted for acute decompensation (the highest prevalence being of 65% in South Asia), while the global 90-day mortality was 58% (highest in South America—73%) [6]. The same study remarks that the most common etiology for chronic liver disease was alcohol consumption, while the most frequent organ failure was renal failure.

**Table 1:** *Main ACLF definitions*, adapted from Bajaj JS et al., Am J Gastroenterology 2022 [7]; HE, hepatic encephalopathy; INR, international normalized ratio; PA, prothrombin activity.

NACSELD	EASL-CLIF	APASL
At least 2 severe extrahepatic organ failures: Shock HE grades III/IV Replacement renal therapy Mechanical ventilation	Patients with cirrhosis Acute decompensation, organ failure, high short-term mortality Organ failure: brain, kidney, liver, respiratory system, circulation, coagulation;	Acute hepatic insult: Bilirubin ≥ 5mg/dL Coagulopathy (INR ≥1.5/PA ≤ 40%) Complicated by ascites and/or HE in < 4 weeks Diagnosed/undiagnosed with chronic liver disease/cirrhosis High 28-day mortality *Extrahepatic organ failure not required*

## 2. Diagnosis and Prognostic Scores

The diagnosis of ACLF is made on diagnostic criteria included in the up mentioned definitions, thus there are no universal criteria for ACLF diagnosis. Depending on the used definition of ACLF, some patients might not be diagnosed even if they fulfill the ACLF criteria using another definition and not be treated accordingly. Thus, standardization of ACLF definition and diagnosis is an unmet need that should be solved in the near future, in order that all ACLF patients be diagnosed and managed properly worldwide.

Depending on the ACLF definition we use, different ACLF clinical phenotypes emerge each with distinctive cause for end-stage liver disease, trigger factor for ACLF, and prognosis [8].

Since the recognition of ACLF as a distinctive syndrome with no universal definition and diagnostic criteria, comparison studies between definitions have emerged. When compared to the APASL definition, the EASL definition of ACLF showed that it can identify more ACLF patients, but in terms of high short-term mortality the results were similar [9]. A comparison between the ACLF definitions of EASL, NACSELD and APASL related to prognosis showed that the EASL definition for ACLF made a better prognosis for mortality at 28 and 90 days [10], while another study concludes that using different diagnostic criteria ends with different prognosis in ACLF patients, which can affect the clinical management of the patients [11]. The first step for a global definition of ACLF can be considered the “Kyoto Consensus” which opens the path for discussions and collaboration between international societies in order to reach a consensus in the future [12].

The CLIF-C ACLF score includes hepatic and extrahepatic organ failures, the number of white blood cells and age and demonstrated better results in predicting mortality and 28 and 90 days compared to the Model for End-Stage Liver Disease (MELD) and MELD- Na score [13]. Also, in the PREDICT study, the CLIF-C ACLF score demonstrated a better prognostic accuracy when compared to MELD, MELD-Na, and Child-Pugh scores [14].

## 3. Precipitating Factors

Over time, several precipitating factors of ACLF were identified, the most common being bacterial infections, followed by severe alcoholic hepatitis, gastrointestinal bleeding with shock and toxic encephalopathy. While the majority of patients with an identified precipitating factor (more than 95%) had a proven bacterial infection or severe alcoholic hepatitis, in 35% of the patients no trigger factor was identified. The presence of more precipitating factors has a cumulative effect as regards the disease course and outcome [15].

Also, the prevalence of trigger factors of ACLF varies worldwide. In China, the main precipitating factors for ACLF are hepatitis B virus infection, followed by bacterial infections [16].

Bacterial infections in patients with cirrhosis are associated with increased mortality [17], while in ACLF patients are the main precipitating factor being identified in approximately 40% of patients. Almost half of ACLF patients with no infection at diagnosis develop a bacterial infection in the first four weeks from diagnosis [18]. Bacterial infections in ACLF patients are associated with a worse clinical course and a lower rate of survival at 90 days compared to non-infected ACLF patients. The most common bacterial infections in cirrhotic patients are spontaneous bacterial peritonitis, urinary tract infections, pneumonia, soft tissue infections, and skin infections [19]. It should be emphasized that infected cirrhotic patients might not have the classic infection symptoms, rarely developing fever and might have “normal” white blood cell despite having an infection [20]. So, a level of suspicion with a screening for possible infection should be made in every admitted patient with decompensated cirrhosis, in order to prevent the ACLF development.

Alcoholic hepatitis was identified as the second most common precipitant of ACLF [15]. The prevalence and severity of ACLF are increased in active alcohol consumers with acute decompensation of cirrhosis compared to the other patients [21]. Patients with alcoholic hepatitis can associate bacterial infections in up to 20% of cases [22]. Also, alcoholic hepatitis is characterized by systemic inflammation that might trigger organ failure. Besides the frequent association with bacterial infections, alcohol consumption is also associated with increased intestinal permeability and bacterial translocation [23]. The systemic inflammation, risk of infections and immune failure cand determine organ failure, with poor prognosis and high rate of mortality [21].

In order to improve the prognosis of ACLF patients, early diagnosis and treatment of bacterial infections is necessary. Studies show that infected patients have a worse prognostic and greater mortality with each hour delay in antibiotic administration [24].

Patients with ACLF should go through an extensive set of investigations for the most common trigger factors in order to identify and treat the disease, while rare precipitating factors should be investigated depending on each individual case presentation [1].

## 4. Organ Failure

In a study of 507 cirrhotic patients admitted with infection, it was reported that 55.7% developed hepatic encephalopathy stage III–IV, 17.6% shock, 15.1% needed renal replacement therapy, and 15.8% required mechanical ventilation [5]. Patients with two or three organ failures had a higher mortality rate at 30 days. Patients with four organ failures had a survival rate of 23% at 30 days compared to 96% in patients with no organ failure.

A larger European study shows that liver and kidney failure are the most frequent organ failures, followed by coagulation, cerebral, circulatory, and respiratory failure [20].

Each major international organization dedicated to liver study developed different score systems in order to evaluate the organ failures in ACLF patients. European researchers, as well as Chinese ones, are using the CLIF-C organ failure (OF) system which evaluates the function of liver, kidney, brain, coagulation, circulation, and respiration, through different clinical and biochemical factors [1]. The CLIF-C OF score has been validated in many studies [23] and is recommended to be used recurrently in order to evaluate the risk of mortality at 28 days [1].

The NACSELD score is made by the number of organ system failures and ranges from 1 to 4, with ACLF being defined by a score of 2 or more [25]. When comparing CLIF-C OF score with NACSELD score, studies have shown that using the NACSELD criteria a significant number of patients with ACLF diagnosis based on CLIF-OF might not be identified [26].

APASL researchers developed a score based on evaluation of brain function, serum levels of bilirubin, coagulation (international normalized ratio or prothrombin time), creatinine, and lactate levels, named AARC score [4]. The score is designed to evaluate the severity of ACLF, not to define it. Studies comparing the CLIF-OF score with the APASL criteria for ACLF diagnosis have shown that the last ones might underrate the presence of ACLF [27].

## 5. Pathophysiology

**Figure 1:**
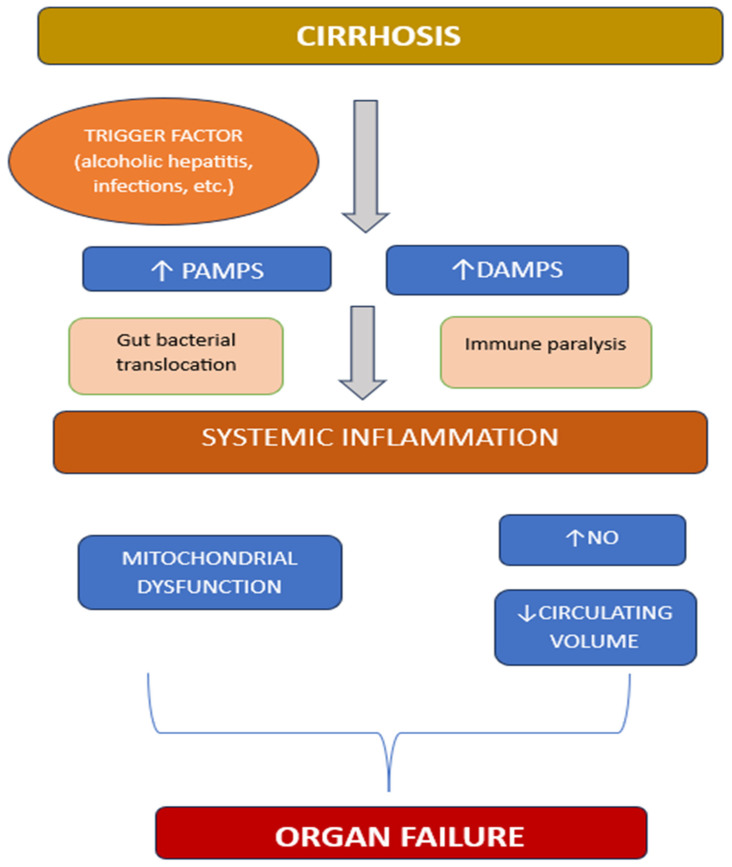
*ACLF pathophysiology*; PAMPs, pathogens-associated molecular patterns; DAMPs, damage-associated molecular patterns; NO, nitric oxide.

Even though the exact mechanism of ACLF syndrome is not completely understood yet, we all agree that systemic inflammation has a fundamental role in the development of ACLF. Alongside systemic inflammation there is an altered immune response characteristic for cirrhotic patients that also contributes to the ACLF process (Figure 1).

The first large studies on ACLF patients showed a higher level of C-reactive proteins levels, as well as higher levels of leukocytes in these patients compared with patients who did not meet the ACLF criteria [20]. At the same time, the leukocyte level proved to be an independent predictor of mortality.

Pathogen-associated molecular patterns (PAMPs) and damage-associated molecular patterns (DAMPs) seem to activate an excessive systemic inflammatory response while there is an altered immune response as well [28]. PAMPs are derived from pathogen organisms, one of the most important ACLF trigger factors, and activate signaling pathways by binding to specific receptors. The same receptors can be activated by DAMPs derived from injured hepatic cells in patients with alcoholic hepatitis, hepatitis B flare, or toxic hepatic injury. The final result is an increased level of inflammatory molecules like interleukins, tumor necrosis factor α and interferon β [29].

Systemic inflammation is a complex process that requires energetic substrate in order to unfold, while the metabolic cost may be implied in the organ failure development [30]. A study that targeted the study of metabolome in cirrhotic patients, with or without ACLF, identified 38 distinctive blood metabolite fingerprints for ACLF that were linked to mitochondrial dysfunction [30]. The level of fingerprint was correlated with the level of inflammatory markers.

The energy required for the inflammatory process is derived from glucose, amino acids, and fatty acids that are released in high amounts secondary to glycogenolysis, proteolysis, and lipolysis [31]. Also, there seems to be a mitochondrial dysfunction with an energetic switch from oxidative phosphorylation to glycolysis and a reduce fatty acid catabolism [15].

At the same time, there is a high level of nitric oxide secondary to systemic inflammation that decreases the systemic vascular resistance and reduces the circulating volume with activation of endogenous vasoconstrictors in response [28]. Also, there is tissue hypoperfusion due to reduced cardiac contractility with an associated reduced cardiac output induced by nitric oxide.

Cirrhosis-associated immune dysfunction (CAID) includes alterations of the immune response in a patient with end-stage liver disease [32]. In ACLF patients, CAID is characterized by immune paralysis, an increased risk of infection and a worse outcome. When compared to patients with severe sepsis, ACLF patients demonstrated to have a similar degree of cellular immune depression which can be associated with a worse infection-related prognosis [33]. Also, the increased intestinal permeability in cirrhotic patients favors bacterial translocation which cand trigger or maintain the systemic inflammation [8].

## 6. Management

### 6.1. General Management

Brain failure defined as hepatic encephalopathy (HE) grade III or IV is the only organ failure with similar criteria for definition alongside ACLF definitions made by the most important international societies. ACLF patients with hepatic encephalopathy have a worse prognosis when compared to patients with HE but without ACLF criteria [34]. Besides the already well-known precipitating factors of HE, a new class of medication might emerge as a precipitating factor. Proton pump inhibitors (PPIs) are prescribed in approximately 60% of cirrhotic patients, with nearly just a half having a documented indication [35]. Some studies show that PPI treatment is linked to a higher risk of hepatic encephalopathy [36], but the number of studies is small, so a clear conclusion cannot be made yet regarding the risk of hepatic encephalopathy and PPI treatment.

Management of patients with HE grade III–IV implies protection of the airways in order to avoid aspiration, exclusion of other causes of altered mental status, identification and treatment of precipitating factors, and empirical therapy for HE [7].

Kidney failure is the most common organ failure among ACLF patients [6]. It should be managed according to current guidelines [37], although the majority of guidelines and recommendations for cirrhotic patients with renal failure are reserved for hepato-renal syndrome (HRS). Vasoconstrictors like terlipressin was demonstrated to be efficient in the treatment of cirrhotic patients with HRS type 1, but adverse events should not be neglected [38]. The association of terlipressin with albumin in the treatment of hepato-renal syndrome was more effective in improving the renal function when compared to albumin alone [39]. For patients who do not respond to proper therapy, liver transplant should be considered with renal replacement therapy used as a bridge to transplant [7].

Respiratory failure is managed either by oxygen administration or by mechanical ventilation in intensive care units. The greatest concern regarding mechanical ventilation is related to ventilator-associated pneumonia (VAP). However, prophylactic antibiotic administration in order to prevent VAP is not recommended in current guidelines [7].

Circulation failure requires administration of pressor support, despite the differences in the definition of this organ failure in the international ACLF guidelines. The NACSELD guideline recommends norepinephrine as the vasopressor of choice in ACLF patients [7].

Coagulation failure is often defined in clinical practice by the international normalized ratio (INR) with transfusions made depending on alteration of coagulation parameters. Laboratory coagulation alterations are common in patients with end-stage liver disease and studies show that thromboelastography (TEG) can assess coagulation better than INR and platelet count, avoiding unnecessary blood product transfusions [40]. Otherwise, transfusions in ACLF patients with coagulation abnormalities are not normally recommended, apart from patients with bleeding or a scheduled invasive procedure [7].

**Figure 2:**
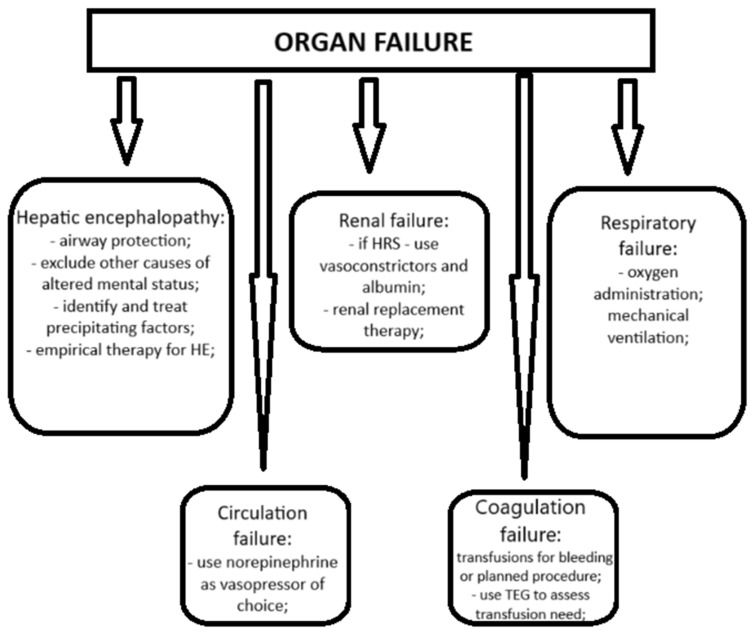
*Management of organ failure in ACLF*, adapted from Bajaj JS et al., Am J Gastroenterology 2022 [7]; HE, hepatic encephalopathy; HRS, hepatorenal syndrome; TEG, thromboelastography.

Management of organ failure in ACLF patients is shown in Figure 2.

As bacterial infections are the most common precipitating factor of ACLF, choosing antibiotic therapy is very important. Local epidemiology of bacterial infections and local resistance to antibiotics are cornerstone for treatment options, besides etiology, infection severity, and how it was acquired. Delay in antibiotic administration, as well as inadequate antibiotic therapy are associated with increased mortality in cirrhotic patients [41]. For patients with ACLF and suspected bacterial infection, a complete infection workup should be carried out as soon as possible, alongside with broad-spectrum empirical antibiotic therapy [1]. ACLF patients with long ICU admission are at risk for fungal infections which are a contraindication for liver transplant. Patients with septic shock and risk for fungal infections should receive antifungal therapy until fungal infection is excluded [42].

Regarding alcoholic hepatitis and ACLF treatment, current recommendations are to use corticosteroids with careful surveillance for infections. The EASL guidelines for ACLF do not recommend the use of corticosteroids in patients with uncontrolled infections or ACLF grade 3 [1]. Studies show that only corticosteroids reduce the risk of short-term mortality in patients with severe alcoholic hepatitis [43]. For patients with contraindication for corticosteroid treatment, pentoxifylline is an option.

### 6.2. Specific Therapies

There is no specific treatment for ACLF at this moment, the entire management of these patients relies on treating precipitating factors, providing organ support, treating associated complications, and evaluating patients for liver transplantation. Several therapies have been studied, but with no positive outcome in regard to survival.

Currently, the definitive treatment for ACLF is liver transplant (LT), demonstrating a high rate of survival post-LT [44]. Patients should be managed by a multidisciplinary team with focus on treating the precipitating factor, supporting organ failure, treating sepsis and evaluating the possibility for liver transplant [7]. Frequently, ACLF patients require admission in the intensive care units (ICU) for organ failure support.

Intravenously administered albumin should be used only in patients with clear indication like preventing renal failure in patients diagnosed with spontaneous bacterial peritonitis (SBP) or in patients that need large volume paracentesis, but not in cirrhotic patients with other infections than SBP in order to prevent organ failure [7].

Artificial extracorporeal liver support systems like molecular adsorbent recirculating system (MARS) or the Fractioned Plasma Separation and Adsorption (Prometheus) have been studied for the treatment of ACLF [45]. While these artificial liver support systems accomplish the detoxifying function of the liver, the bioartificial extracorporeal liver support systems cand perform both the detoxifying and the synthetic functions as well with the mention that the prerequisite of hepatic cells (human or porcine) is significant. The studies on these systems in ACLF patients are small, showing a reduction in serum bilirubin and grade of HE [46,47], but no improvement in survival [48], as such they are not included in current guidelines as a treatment for ACLF patients. Also, a comparative study between standard medical therapy and MARS in ACLF patients showed no difference in survival at 28 and 90 days between the two groups [46].

Granulocyte colony-stimulating factors (G-CSF) were compared to placebo and standard medical therapy (SMT) in a study on ACLF patients. The results were promising, showing a rate of survival at 60 days of 66% in the G-CSF group compared to 26% in the placebo group [49]. Another study showed similar results in patients with hepatitis B virus-associated ACLF [50]. Contradictory results were obtained in the GRAFT study that included 176 ACLF patients, with no difference in survival at 360 days between G-CSF therapy and SMT [51]. Although the results seem to be promising, the number of studies and patients included is low and future studies are needed in order to confirm or infirm the beneficial effects of these therapies in ACLF patients.

Mesenchymal stem cell therapy might emerge as a new therapy in the next years, showing beneficial effects in patients with chronic liver disease and ACLF [52].

Multiple therapies are currently under investigation in animal or human models, targeting different pathways of the pathophysiological process of ACLF which is a dynamic process with multiple variables, with the hope that at least some of them will be the future ACLF treatment [53].

Toll-like receptors (TLRs), a family of transmembrane pattern recognition receptors that can recognize different structural components of bacteria, viruses or fungi (PAMPs), play an important role in both innate and adaptive immunity [54]. The first discovered TLR was toll-like receptor 4 (TLR 4) that mainly binds to lipopolysaccharide (LPS) and mediates tissue injury in liver failure [55]. TAK-242, a TLR4 inhibitor, showed an improvement in survival and reduced liver and kidney damage in rodent models [56]. Unfortunately, a phase 2 clinical trial was shut before recruiting patients [54]. A combination of TAK-242 and G-CSF showed promising results in ACLF mouse models with inhibition of inflammation and promoting of hepatic regeneration, which can be a future therapeutic option in ACLF patients [57].

Receptor-interacting protein kinase 1 and 3 (RIPK1 and RIPK3) play an important role in necroptosis, a form of scheduled necrosis that shares the same receptors as apoptosis, but ends up with cell swelling, rupture, and release of DAMPs [55]. In HBV-ACLF patients, the serum levels of RIPK3 were significantly higher compared to non-ACLF patients and were associated worse outcomes [58]. Also, in animal models of ACLF, it was demonstrated that plasma levels of RIPK1 cand predict the risk of mortality and progression to ACLF [55]. In the same study, the pre-use of RIPK1 inhibitors showed improved liver and kidney functions, with a reduced liver cell death.

Hepatic natural killing (NK) cells increase hepatocyte apoptosis in HBV-ACLF patients [59]. The potassium channel tetramerization domain containing 9 (KCTD9) protein is highly expressed in hepatic and peripheral NK cells of HBV-ACLF patients and might play an important role in NK cell function regulation [60]. Inhibiting KCTD9 determined an alteration of NK cell function in mouse model, with reduced cytokine production and cytotoxicity, as well as improved liver function and prognosis [61].

Interleukin-22 (IL-22) plays an important role in liver regeneration [62]. A recombinant fusion protein made of two human molecules of IL-22 linked to an immunoglobulin constant region (IL-22Fc) was studied in mouse models of ACLF, with improving survival by reversing the anti-regenerative pathway to the pro-regenerative one [63]. Another form of IL-22Fc named F-652 was studied in a phase 2 clinical trial in patients with alcoholic hepatitis. The results are promising, with a reduction in inflammation markers, increase in liver regeneration markers, decrease in MELD and Lille score, and no serious adverse effects [64].

Many other molecules were studied with no beneficial effect on survival, while other molecules show promising results in preclinical studies, but human studies are needed in order to confirm results [53].

### 6.3. Liver Transplant

Liver transplant (LT) is for now the only curative treatment for ACLF, with 80% rate of survival at one year post LT [65].

Unfortunately, LT lists do not take into consideration the ACLF condition and these patients are included using the traditional scores like the Model for End-Stage Liver Disease (MELD) or MELD Na, despite the fact that ACLF is associated with short-term significant mortality [66]. These traditional scores cannot assess the real mortality rate in ACLF patients [66]. Studies show that a lower MELD score in a ACLF grade 3 patients is associated with a significant higher mortality than a higher MELD score a patient without ACLF [67]. At the same time, a patient with a higher number of organ failures has a higher rate of mortality at 30 days, while an early LT gives a 1 year survival rate almost similar between a patient with three OF(s) and one with five to six OF(s) [68]. So, it seems that the classic criteria for inclusion on the LT waiting-list underestimate the mortality risk in ACLF patients, while these patients do not have fair access to the LT waiting lists due to current allocation systems [69].

In order to solve these inequity problems of inclusion criteria, the EASL guidelines recommend pilot programs of prioritization for ACLF grade 3 patients on the waiting list [1], with the first program being successfully introduced in the United Kingdom [70]. Also, the Spanish Society of Liver Transplantation recommends the prioritization of ACLF patients on liver transplant lists given their poor prognosis and high short-term mortality that might be underestimated by the MELD score [71]. Another solution would be the development and validation of better prognostic scores for ACLF patients regarding inclusion on liver transplant lists in order to make prioritization more efficient. At present, the CLIF-C ACLF score outperforms MELD, MELD-Na, and Child-Pugh scores in terms of short and long-term mortality [72,73]. A score that includes both ACLF grade and MELD score has been developed, but needs validation in large prospective studies [74].

Despite the undeniable benefit of survival, LT is also associated with complications [68] and mortality, especially in ACLF grade 3 patients [67]. Factors like multidrug-resistant infections, renal replacement therapy and pre-LT arterial lactate level > 4 mmol/L are post-LT mortality predictors [65].

### 6.4. Nutrition

Current guidelines recommend a daily caloric intake of 35–40 kcal/kg/body weight/day with a total of 1.2–2.0 g/kg body weight/day of protein intake in ACLF patients [7]. For patients that are unable to meet their nutritional intake by oral route, enteral, or parenteral feeding are recommended. The belief that protein restriction in HE patients might be beneficial is still popular, despite no proven clinical evidence [75]. Studies show that ACLF patients have inadequate nutritional intake, with impairment of the intestinal barrier, while nutritional support was associated with a lower mortality rate at 28 days [76]. ACLF patients had a decrease in skeletal muscle and fat content, while the coccus-bacillus ratio and secretory immunoglobulin A were increased. Also, an association between sarcopenia and both short and long-term mortality in ACLF was found [77].

### 6.5. Future Directions

Over the past years there was a significant progress related to our understanding of ACLF mechanisms and precipitating factors, but many questions remain unanswered.

The variability of ACLF definition around the world might exclude some patients with ACLF outside diagnostic criteria and thus their management would not be the same as for another ACLF patient. Thus, we need global standardization of definition, diagnostic criteria, as well as clinical management in order that all ACLF patients benefit the same medical care.

We also need prognostic scores for patients that are at risk of ACLF development, in order to intensify the research for precipitating factors and avoid ACLF development, as it is associated with high mortality, despite proper management. We expect that biomarkers will soon be used in clinical settings for ACLF patients, as there are studies that identified metabolites of microbial origin that are associated with ACLF and death for cirrhotic inpatients or within 30 days [78].

Bacterial infections are a common trigger of ACLF, as well as a common complication in the course of ACLF evolution. Early screening and diagnosis of infection in ACLF patients can improve the prognosis. Besides infection screening, biomarkers like C-reactive protein and procalcitonin are frequently used in the general population for bacterial infection diagnosis, but with limited use in ACLF patients [79]. Novel scoring systems for diagnosing bacterial infections in ACLF patients are emerging and show promising results [80]. Even antibacterial treatment can be guided by specific biomarkers in order to avoid drug-resistance, the unnecessary use of medication, and antibiotic-related complications [81].

In many cases, ACLF patients need clinical monitoring and organ support in intensive care units (ICU). Due to high-mortality associated with ACLF despite organ support, ICU admissions might be delayed or considered inappropriate, despite the fact that survival rates are increasing in cirrhotic patients admitted in ICU [82]. Studies show that even severe ACLF grade at ICU admission does not alter the outcome of 1-year transplant-free survival after hospital discharge [83]. ACLF patients need to be managed in a multidisciplinary team, including intensive care doctors, such that the prognostic pessimism related to ICU admissions of these critical patients should be changed over time, especially as the ICU outcomes of ACLF patients is comparable with the outcomes of patients without chronic liver disease [84].

With the advances in pathophysiology understanding of ACLF, targeting therapies have been studied, with encouraging results in preclinical trials, but with failure in human trials. One of the explanations can be related to animal models that cannot achieve the complexity of human models. So, we need more complex animal models in order to test new therapies, even though this would lead to a prolonged research progress. On the other side, due to a complex pathogenesis, we might consider targeting more pathways of the ACLF process in order to obtain significant clinical results [53].

The liver is the first organ to interact with gut microbiome products and their role in liver diseases might be more important than previously thought [85]. Gut microbiome might play an important role in the development of ACLF by translocation of gut microbiota through increased intestinal permeability and portal hypertension [85]. Metabolites derived from gut microbiota were associated with ACLF development and mortality, suggesting the role of gut microbiota in ACLF development but also the possibility of using gut microbiota as a biomarker [78]. Quantitative metagenomics analyses revealed a total of 75,245 genes that predominate in cirrhotic patients compared to healthy individuals and biomarkers specific to liver cirrhosis were revealed, a tool that can be used for diagnosis in the future [86]. Therapies targeting gut microbiota and gut-bacterial translocation are currently used or are under evaluation. Rifaximin, a poorly absorbed antibiotic that is used to prevent recurrent hepatic encephalopathy, demonstrated improved survival, and a lower number of portal hypertension related complications on long-term administration [87]. Also, rifaximin might act as a gut microbiota regulator which may increase the number of probiotic bacteria and reduce the number of harmful bacteria [88]. Probiotics might have a positive outcome in ACLF patients, improving liver and immune function and reducing the proliferation of harmful intestinal bacteria [89], but larger studies are needed.

While treatment with simvastatin reduced inflammation and liver damage in ACLF animal models [90], in humans, treatment with statins is associated with a reduced risk of ACLF development [91] and decreased gut microbiota dysbiosis [92]. Non-selective beta-blockers are used for primary and secondary variceal bleeding, but they seem to have other beneficial effects such as reducing systemic inflammation by reducing bacterial translocation [93].

Fecal microbiota transplantation (FMT) was evaluated in thirty-three patients with ACLF and severe alcoholic hepatitis and the result were promising, with improved survival at 28 and 90 days and remission of hepatic encephalopathy and ascites [94]. In patients with severe alcoholic hepatitis, FMT was associated with a reduced number of infections, episodes of HE, lesser ascites and alcohol relapse [95]. FMT might impact the microbiota of liver diseases by reducing systemic inflammation and modulating gut dysbiosis with positive outcomes, but larger studies are needed to confirm this preliminary results [96]. Also, manipulation of gut microbiota can be made by a non-antibiotic product that has the capacity to absorb and clear toxins, a novel-engineered, orally administered carbon bead named CARBALIVE [97]. Gut microbiome-targeted therapies might be a future therapeutic option for ACLF, but more research in this field is needed.

Patients with severe forms of ACLF should be evaluated for liver transplant, as it is the only curative treatment at present [1] and showed significantly higher survival rates compared to no transplant [98]. Given the fact that we all know that ACLF is a dynamic and progressive syndrome with an associated high-mortality, and that there are data suggesting that some ACLF patients might be misclassified on the waiting list by MELD and MELD-Na score, a new question arises: should we have prioritization lists for ACLF patients? [68,99]. The United Kingdom (UK) started a pilot program that prioritizes liver transplantation for ACLF patients with good results [100].

## 7. Conclusions

The heterogeneity of ACLF definitions might be an expense for many ACLF patients, excluding them from proper treatment and inclusion on liver transplant waiting lists. A global consensus of definition and diagnostic criteria of ACLF is mandatory in the future and a collaboration between the international experts and societies can be a solution, based on current knowledge and research. Also, understanding ACLF mechanisms opens the door for novel therapeutic options that are an unmet need for this dynamic syndrome characterized by high short-term mortality. The complexity of ACLF syndrome slows down the pathway for novel therapeutic agents and biomarkers, while individualized treatment might be the future key for these patients. We need more phase two clinical studies using the molecules that already showed promising results, and we also need to encourage researchers to continue their work despite unfavorable results. The only curative treatment at present—the liver transplant—is shaded by the shortage of liver donors, difficult access, and associated complications, as well as the high costs. The severity and high-risk short-term mortality associated with ACLF must be taken into consideration by prioritization lists, as well as the use of special prognostic scores. Until new therapies are available, we rely on diagnosis, prevention and support of organ failure, as well as liver transplants.

## Data Availability

Not applicable.
